# So Many Faces, Phases, and Facets, Sickness Behavior Beyond Disciplines

**DOI:** 10.3389/fpsyt.2021.630331

**Published:** 2021-02-25

**Authors:** Jan Pieter Konsman

**Affiliations:** Aquitaine Institute for Integrative and Cognitive Neuroscience (INCIA) UMR CNRS 5287, University of Bordeaux, Bordeaux, France

**Keywords:** biopsychosocial medicine, disease, health, illness, interdisciplinarity, sickness behavior

## Abstract

Animals, including human beings, modify their behavior when they fall sick. Interestingly, sociology, biology, and psychology have at different times in their history developed constructs of illness or sickness behavior. The aims of the present paper are to consider sickness behavior in animals and humans and to evaluate to what extent the notions of sickness behavior would allow for interdisciplinary research. After distinguishing disease, illness, and sickness, the case will be made that illness behavior and sickness behavior can be considered heuristically as synonyms given the existence of some fluidity between the notion of illness and sickness. Based on this, different faces, phases, and facets of sickness behavior will be presented before addressing the question of how integration of constructs of sickness behaviors would be possible across biology, medicine, psychology, and sociology. It is concluded that interdisciplinary research on sickness behavior between biology, psychology, and sociology is possible and called for with regard to constructs, methods, and explanations, while keeping in mind differences in perspectives, for example between acute and chronic sickness behavior.

## Introduction: Sickness as an Expression of Health and Disease

Even though philosophers of medicine may recently have seemed to agree to disagree on the possibility of consensus definitions of health and disease (1), the American Veterinary Medical Association has approved in 2008 the “One Health” imperative to promote collaboration between several disciplines “to attain optimal health for people, animals, and our environment” ((2), p. 13). This global perspective of health begs the question of how health relates to sickness and disease. One of the best illustrations of a face of sickness is *The sick child* painted several times by Edvard Munch between 1885 and 1926 that features his sister suffering from tuberculosis. Since then, there have been many debates, both in society and within academia, about what health, sickness, and disease would, could, or should be. This is important to keep in mind when discussing sickness as it is often positioned or described relative to health and disease. Here we will first consider a few descriptions of sickness in relation to health and disease before discussing differences in more detail.

*The normal and the pathological* published by the French physician and philosopher of medicine Georges Canguilhem during WWII is an interesting starting point because this scholar distinguished “the experience of being sick” from disease, alluded to changes in behavior during sickness, and linked the possibility “to fall sick” to “good health.” According to Canguilhem, medical science should not aim to generate a general disease concept but rather “determine what are the vital phenomena with regard to which men call themselves sick” ((3), p. 122). He further pointed out that different conceptions of disease based on deficiency, infection, or dysfunction on the one hand, and “the experience of being sick” on the other hand have in common the idea of “an internal struggle between opposing forces” ((3), p. 41). Within this general framework, Canguilhem drew attention to changes in “the sick person's personality” in that he or she can have “reactions which never turn up in the normal subject in the same form and in the same conditions,” but that “are not the result of an impoverishment or diminution” ((3), p. 184). Such changes in behavior, which are readily observed during inflammation when “the anti-infectious defense is mobilized,” implied for him that “[t]o be in good health means being able to fall sick and recover” ((3), p. 199).

Some years after Canguilhem, the American internist and psychiatrist George Engel proposed to link different levels of organization and various responses observed after bacterial infection. At the tissue level, this results in an inflammatory response while at the level of the whole organism it can give rise to a profound alteration of physiology (4). However, Engel noted that “we also see psychologic defenses, illustrated by such phenomena as regression, increased dependence, withdrawal of interest in the outer world” ((4), p. 55). Finally, he remarked that institutionalized medical care and social help can be solicited (4). In his later work, Engel tried to articulate these different responses and he put forward that “[a]s various systems of the body are brought into action to cope with the local process [of infection], this eventually influences total behavior” and that with the activation of “central neuro-humoral systems … must … come an impact on systems of internal perception of the mental apparatus indicating the change in the bodily status” ((5), p. 50). In turn, this is perceived “as an affect, a general sense of malaise, fatigue, restlessness, uneasiness, [or] vague anxiety” ((5), p. 50). In his subsequent biopsychosocial model of medicine, Engel argued that disease is a term that refers to objective phenomena, while other terms have been used to express personal experience “associated with impairment or discomfort” ((6), p. 130). He then insisted that “[p]sychological and social factors” are important “in determining whether and when patients with the biochemical abnormality of diabetes or of schizophrenia come to view themselves or be viewed by others as sick” to make the point that “boundaries between health and disease, between well and sick, are far from clear and never will be clear” ((6), p. 132). He thus indicates that it is not necessarily because an individual has been diagnosed with a disease by a physician by establishing a plausible cause for some clinical findings that that person feels sick or is considered sick by his environment. An attempt to provide some more clarity on the distinctions between disease, illness, and sickness will nevertheless be undertaken in the next section.

### Distinctions Between Disease, Illness, and Sickness

In the mid-1970s, 2 years before Engel's proposal for biopsychosocial medicine, the philosopher of medicine Christopher Boorse put forward a distinction between disease and illness. According to him, disease “applies indifferently to organisms of all species” and “it is to be analyzed in biological … terms” ((7), p. 56). Instead, illness is a disease for which “its owner deserv[es] special treatment and diminished moral accountability” ((7), p. 56). That same year, the general practitioner and professor of medicine Marshall Marinker provided a further distinction between disease as “a pathological process, most often physical,” illness as “a feeling, an experience of unhealth which is entirely personal” and sickness as “the external and public mode of unhealthy” ((8), pp. 82–83).

The philosopher of medicine Bjørn Hofmann has more recently noted that the notions of disease, illness, and sickness have been “more strictly defined …, but also fundamentally challenged” in “medical sociology, medical anthropology, and philosophy of medicine” ((9), p. 651). Thus, the sociologist Talcott Parsons already indicated in 1951 four societal expectations “relative to the sick role,” namely, (1) “the exemption from normal social role responsibilities,” (2) “the sick person cannot be expected by “pulling himself together” to get well,” (3) “the state of being ill [i]s itself undesirable with its obligation to want to “get well,” and (4) “the obligation … to seek technically competent help, namely, in the most usual case, that of a physician and to cooperate with him in the process of trying to get well” ((10), pp. 436–437). Later, the sociologist Andrew Twaddle suggested that adaptation could be a more appropriate characterization of the sick role than Parson's deviance framework ((11), p. 260, p. 270). Hofmann has argued that Twaddle's own triad of disease, illness and sickness is related to the World Health Organization's definition of health as “a state of complete physical, psychological and social well-being” ((9), p. 655) and thus reflected a perspective different from that of Parsons. These considerations led Hofmann to his own definitions according to which disease corresponds to “negative bodily occurrences as conceived of by the medical profession,” illness to “negative bodily occurrences as conceived of by the person himself” and “sickness [to] negative bodily occurrences as conceived of by the society and/or its institutions” ((9), p. 657).

While it is understandable that there has been substantial interest in studying disease, illness, and sickness affecting human beings, it is also important to keep in mind that animals other than human beings can fall ill and are diagnosed with diseases by veterinarians. Animals have been used as work force, life stock, experimental subjects, and pets by man, and it is in these relationships that animals have been considered as victims of disease and sometimes as patients (12). In the 19th century, the physician and pathologist, Rudolf Virchow emphasized “that there is no scientific barrier, nor should there be, between veterinary medicine and human medicine” as “the experience of one must be utilized for the development of the other” (cited in Bollinger (13), p. 7). However, even though it is impossible to have access to their experience, humans have long recognized sick animals, often because they were not performing the behaviors of interest to man, and tried to identify diseases. It has been argued that the existence of two roles of animals, namely, as subjects of veterinary care and as objects of medical and zoological research, has allowed for connections to occur between these fields and to have thus favored the development of the “multifaceted domain of ‘agricultural [and veterinarian] science”' ((12), p. 105, p. 107). After remarking that the behavioral symptoms of disease, such as lethargy and reduced food intake, are not specific to a particular species and referring to studies showing that both fever and reduced food intake can favor survival of infected animals (14, 15), the veterinarian Benjamin Hart proposed at the end of the 1980s the concept of sickness behavior as “a highly organized behavioral strategy” with a “biological basis” ((16), p. 123).

The aims of the present work are therefore to consider sickness behavior both in humans and other animals and to evaluate to what extent the notion of sickness behavior would allow for interdisciplinary research. In the remainder of this introduction, and after having distinguished disease, illness, and sickness, some fluidity between the latter terms will be pointed out to make the case that illness behavior and sickness behavior can be considered heuristically as synonyms. Based on this, the second part of the paper will present different faces, phases, and facets of sickness behavior before addressing the question of how integration of constructs of sickness behaviors would be possible across biology, medicine, psychology, and sociology.

### Some Fluidity Between Illness and Sickness

Interestingly, and after spelling out differences between these terms, Bjørn Hofmann also admitted that there are conditions that are considered disease and illness, but not sickness, others that are deemed disease and sickness, but not illness, and still other conditions that are viewed as sickness and illness but not disease. Among the latter would be fibromyalgia and chronic fatigue syndrome (9), in which individuals feel ill and are often recognized as being sick by their immediate environment and more or less by the societies in which they live, but do not have a disease as long as medical science has not identified plausible causes for these syndromes. In addition, there seems to be some porosity between illness and sickness in the ways they are employed in academia. Indeed, if according to Hofmann illness corresponds to “negative bodily occurrences as conceived of by the person himself” and sickness to “negative bodily occurrences as conceived of by the society and/or its institutions” ((9), p. 657), then it would be hard to understand why Talcott Parsons has used illness and sickness as synonyms, for example, when he wrote that “medical practice may be said to be oriented to coping with disturbances to the ‘health' of the individual, with ‘illness' or ‘sickness”' ((10), p. 429). Another sociologist, David Mechanic, has elaborated on Parsons' sick role by proposing the term illness behavior to describe the ways symptoms can be interpreted differently and result in different kinds by various individuals ((17), p. 189). Thus, illness for Mechanic is not just a state but also a way of coping that determines, in part, if and how the individual appeals to modes of care, including those offered by society (18). Finally, and although the terms “illness” and “illness behavior” can be found regarding animals, the term “sickness behavior,” proposed in 1988 by the veterinarian Benjamin Hart, is most prevalent.

Interestingly, typing “sickness behavior” in the Medical Subject Headings (MeSH) database on PubMed takes one to illness behavior. On PubMed, illness behavior is, since 2009 described as a “[c]oordinate set of non-specific behavioral responses to non-psychiatric illness” that “may include loss of appetite or libido, disinterest in activities of daily living or withdrawal from social interaction” (https://www.ncbi.nlm.nih.gov/mesh/?term=%22sickness+behavior%22). Of note, between 1975 and 2008, prior to the current heading of illness behavior, this term was indexed under “sick role” (https://www.ncbi.nlm.nih.gov/mesh/?term=%22sickness+behavior%22). Importantly, interrogating PubMed with “illness behavior” as a keyword set gives twice as many results as “sickness behavior.” Similarly, searching for “illness behavior” on the American Psychological Association (APA) PsychInfo database results in almost 10 times more articles than searching for “sickness behavior.” This difference between the PubMed and PsychInfo databases may be explained, in part, by the fact that illness behavior has been part of the APA Thesaurus of Psychological Index Terms since 1982 and is defined as “behaviors, attitudes, and emotions exhibited by individuals during the course of a physical or mental illness,” whereas “sickness behavior” is not part of this index. Finally, typing “sickness behavior” as a search topic on the Web of Science (WoS) Core Collection yields more hits than “illness behavior.” This suggests that overall in WoS-indexed articles “illness behavior” and “sickness behavior” are used differently than in articles found on PubMed and PsychInfo. Given this varying and somewhat liberal use of both terms, sickness behavior will be considered heuristically here as a synonym of illness behavior.

A final note of clarification concerns the distinction between syndrome and disease, which are sometimes used “improperly and ambiguously” (19). A syndrome can be defined as “a recognizable complex of symptoms and physical findings which indicate a specific condition for which a direct cause is not necessarily understood” while the term disease is employed when “medical science identifies a causative agent or process with a fairly high degree of certainty” (19). In keeping with the overall distinctions related above and the spirit of a syndrome as a collection of symptoms and physical findings, while recognizing that the causes for many of the disease that are accompanied by illness or sickness behaviors are known, different kinds of syndromes can be distinguished. Thus, (1) an illness-sickness syndrome could be conceived of as a collection of feeling cold, having a fever, fatigue, sleeping more, lower appetite, reduced food intake, wanting to be alone, and not engaging in social activities; (2) a sickness response syndrome would tentatively include all observable or measurable responses like a fever, increased time sleeping, lower food intake and less social interactions; and (3) a sickness behavior syndrome would be a syndrome of behaviorally observable changes, such as sleeping more, reduced food intake and not engaging in social activities.

## Different Faces, Phases, and Facets of Sickness Behavior

Without attempting to give an exhaustive overview of the various authors, periods, and aspects that can be distinguished regarding concepts of illness/sickness behavior, the idea here is rather to highlight some developments in different lines and traditions of research in sociology, biology, and psychology.

### Sociology: The Sick Role and Illness Behavior in Medical Practice

The first line of research has been sociological and comprised the study of the so-called sick role and illness behavior. In 1951, Talcott Parsons considered that in Western societies illness is motivated as it comes with “the exemption from normal social role responsibilities” that may constitute a “secondary gain” ((10), pp. 436–437). Later, Parsons acknowledged that this line of thinking was inspired by the prevalent idea in the 1930s that psychological factors play an important role in somatic disease ((11), p. 258). He made a parallel with “accident-prone people” who are at increased risk of having an accident, but for whom the consequences of such accidents should not be considered from a point of view of motivation to argue that “similar considerations apply to such fields as infections, and … cancer” ((11), p. 260). Finally, Parsons also distinguished acute illness during which the individual is fully engaged “to coping with the state of illness” and chronic illness, which necessitates “only a very partial attention on the part of the patient” ((11), p. 269). David Mechanic also seemed to have put Parsons' early ideas into some perspective by pointing out that some individuals “may be motivated to adopt the sick role to obtain release from various kinds of responsibilities,” but other individuals “who fear the dependence of the sick role or who are suspicious of physicians and avoid seeking medical advice even when serious symptoms appear” ((17), p. 190). Thus, in North-American sociology of the 1950s and 1960s, the constructs of the sick role and illness behavior were clearly linked to motivation but in different ways.

At the end of the 1960s, the South-African physician Issy Pilowsky introduced the notion of abnormal illness behavior to qualify a situation in which the patient does not seem to agree with the physician's diagnosis and proposed solution for the problem that the patient expressed (20). Another point of Parsons' “sick role” that has been discussed is that of the individual's responsibility. The Canadian sociologist Alexander Segall has proposed to distinguish between physical conditions for which Parson's sick role including the lack of responsibility of the ill individual would hold and a psychological conditions for which “the question of personal responsibility arises” ((21), pp. 163–164). The philosopher of science William Bechtel has wondered if, in the light of certain cultural changes that have taken place in Western societies since Parsons' publications of the 1950s, and in particular given the “growing sentiment that in many cases an individual is responsible for being sick,” the concept of the sick role needs to be revised ((22), p. 131).

It is therefore not that surprising to see Pilowsky propose the “Illness Behavior Questionnaire” in the 1980s with subscales assessing hypochondria and the “psychologic versus somatic perception of illness” (23). Around that time, the Mexican-American sociologist Angelo Alonzo judged that even though the concepts of the sick role and illness behavior have motivated numerous studies in medical sociology, this had not led to a better understanding of social behavior in the context of illness and disease (24). His aim was therefore to propose an integrated behavioral model that can be used by different disciplines to further “understanding, assessing and intervening in social behavior surrounding disease and illness” ((24), p. 499). Adopting a “situational-adaption perspective to health and illness,” Alonzo distinguished “everyday, acute, chronic and life threatening” illness behaviors ((24), p. 508). He also seemed to agree with David Mechanic that illness and behavior are not necessarily dependent in the sense that “some individuals seek medical care at the slightest health deviation [and] others must be coerced by law to present themselves for evaluation and treatment” ((25), p. 160). More recently, a plea was made to describe abnormal illness behaviors taking into account types of illnesses and to be generally cautious when using the label abnormal in these cases (26). Finally, some authors have proposed that illness behavior may integrate “lines of research [that] have been concerned with illness perception, frequent attendance at medical facilities, health care-seeking behavior, treatment-seeking behavior, delay in seeking treatment, and treatment adherence” ((27), p. 74).

### Biology: Sickness Behavior as an Adaptive Regulated Response to Infection

The second line of research has been biological and driven by the idea that sickness behavior is an adaptive regulated response to infection. In his 1988 review that introduced the term “sickness behavior” for animals, Benjamin Hart provided a table of infectious diseases affecting domestic animals and human people that have been reported to be accompanied by fever, reduced food intake and behavioral depression (16). As indicated above, he considered that the behavioral symptoms of disease are not specific to a particular species and that fever is an adaptive response favoring survival of infected animals. Accordingly, Hart argued that anorexia reduced the likelihood of an animal engaging in locomotor activity to search for food and thus allowed to preserve the body's energy stocks that are needed to increase body temperature and mount a fever response (16). Similarly, he pointed out that curling up during an infectious disease reduced the surface area of the body and thus attenuated heat loss (16). In addition, Hart related findings indicating that the pro-inflammatory cytokine interleukin-1 (IL-1) not only is an endogenous pyrogen but also reduces food intake and locomotor activity as well as induces sleep in animals and humans (16). Not surprisingly, he concluded that fever and the behavioral symptoms of disease are brought about in a coordinated manner through the action of IL-1 as part of “an evolved disease-fighting strategy” ((16), p. 131).

One of the ways in which Benjamin Hart described sickness behavior was in terms of motivation (16). This question has been picked up and expanded by the French veterinarian Robert Dantzer who hypothesized that reduced appetite, reduced activities, and social withdrawal typical of sick animals are the expression of changes in motivational priorities. The findings of his group showing that that sickness behavior in rodents, provoked by systemic injection of bacterial lipopolysaccharide (LPS), occurred as a function of external conditions, for example temperature and food availability (28, 29) corroborated the idea that sickness behavior is the expression of a motivational state. It has been also argued that increased sensitivity to pain or hyperalgesia typical of inflammation may be adaptive by avoiding the use of the painful body part and attending to it and should be “viewed as a part of sickness behavior” ((30), p. 96). Given that motivated behaviors are regulated by the central nervous systems, this opened new research avenues proposing several immune-to-brain signaling pathways and brain circuits mediating sickness behavior in the 1990s and 2000s (31). Finally, and over the past decade, various interventionist approaches have allowed to describe neurobiological mechanisms underlying reduced food intake of rodents in response to the administration of bacterial LPS or pro-inflammatory cytokines in quite some detail (32–35).

### Psychology: Sickness Feelings and Cues

Psychology has picked up on the theoretical motivational framework laid down by the sociologists Tascott Parson and David Mechanic and studied the behavior of individuals with chronic medical condition in the context of coping styles. Thus, in adult cancer patients, protective buffering, which includes withholding or denying hiding cancer-related thoughts and concerns (36), was found to be motivated in large part by protection of one's partner (37). While many scholars trained in psychology also participated in the biological approach to sickness behavior by providing their expertise in animal behavioral testing, a third and psychological line of research on sickness behavior emerged after it was shown that the administration of bacterial LPS in humans also induces a transient systemic inflammatory response (38, 39). The first studies addressing LPS-induced sickness behavior in humans concerned phenomena previously established in rodents, namely, increased non-Rapid-Eye Movement sleep, conditioned aversion, and hyperalgesia (40–43). Moreover, and just as animal locomotor activity is reduced after LPS administration, LPS was found to lower walking speed in human volunteers (44). Interestingly, in terms of motivation, LPS injection to healthy volunteers has been reported to decrease “acceptance rates of high-effort options,” but to increase “incentive motivation when the effort is deemed worthwhile” (45, 46). Other early studies have tested cognitive functions, thus also expanding work done in animals, and found that LPS administration decreased memory function (47, 48).

Furthermore, these and follow-up studies assessed emotions and mood, which is notoriously difficult or impossible to do in animals, and reported increased anxiety and depressed mood (47–49). In addition, a mainly feelings-based “sickness questionnaire” has been developed “to assess sickness behavior” in humans (50). Interestingly, recognition of a sick person by non-medical professionals did not require a questionnaire and could be made based on gait and facial cues (44, 51). Although there is a substantial number of articles that have applied functional brain imaging approaches to LPS-injected animals by detecting Fos transcription factors, these approaches are limited by the long-time window between the stimuli of interest and increased Fos expression indicating genomic activation and the fact that they require the animal to be sacrificed (52, 53). With the advent of wider implementation of functional brain imaging approaches in human, like Blood Oxygen Level-Dependent Magnetic Resonance Imaging, stimuli and metabolic activation could be studied in conscious subjects. Such studies have shown increased activation of right inferior orbitofrontal cortex in response to emotional visual stimuli after LPS administration (54) and increased functional connectivity between the left anterior insula and left midcingulate cortex (55). Thus, it has become possible to relate feelings, perception of cues or task performance, and metabolic cerebral activation patterns during sickness.

## Integration of Constructs of Sickness Behaviors Across Disciplines in Biopsychosocial Medicine?

Epidemiology has a respectable historic tract record in pointing out potential causal relationships, for example between smoking and lung cancer, to biomedical disciplines that can mobilize intervention strategies to test the causality of the relationship (56). While sociology has the potential to play a similar role, this turned out to be more complicated perhaps because sociology is overall a less quantitative discipline than epidemiology and because of the increased focus on personal responsibility in disease between the 1950s and 1990s (57, 58). As a result, the influence of sociology on biomedical sciences may have depended more on scientific constructs, concepts, and ideologies. In spite of this, the very term medical sociology has moved from the cover of a book by David Mechanic in 1968 to a presently theory-rich sub-discipline of sociology (58). Finally, it has also been argued that epidemiological reasoning has been modified by sociological evidence, for example related to the notion of stress (59). Regarding illness/sickness behavior, it seems indeed that sociology was first to develop constructs under that banner. However, it does not seem to be the case that this was then passed on or seeped through to psychology and biology. Instead, while the construct of illness/sickness behavior developed in sociology inspired part of psychology, biology appeared to have developed its own construct, which then, in turn, influenced another part of psychology.

### Biopsychosocial Medicine

Given that concepts of illness/sickness behavior have been developed in biology, psychology, and sociology, one may wonder to what extent these constructs are similar or compatible and what, if any, role illness/sickness behavior plays in the so-called biopsychosocial model of medicine. Indeed, George Engel, who proposed the biopsychosocial model of medicine in the 1970s expressed the hope that the study of all contributing factors would allow to explain “why some individuals experience as ‘illness' conditions which others regard merely as ‘problems of living”' ((6), p. 133). He envisioned his “biopsychosocial concept of disease” as an approach allowing for the study of disease and medical care as interconnected phenomena ((6), p. 134). Engel emphasized that the first source of clinical information for a physician is the patient between his or her reported feelings, sensations, and thoughts and observable behavior and signs and that clinical study starts “within a two-person system, the doctor-patient relationship” ((60), p. 108). He thus encouraged physicians to acquire information and skills from “the psychosocial areas” in order “to have a working knowledge of the principles, language, and basic facts of each relevant discipline” ((60), p. 121). Moreover, for a biopsychosocial physician and in the interest of the patient, Engel recommended that “higher system level occurrences must be approached with the same rigor and critical scrutiny that are applied to systems lower in the hierarchy” ((60), p. 121). The reference framework here is systems theory according to which different levels of organization are hierarchically connected “so that change in one affects change in the others” ((6), p. 133). Within this framework, Engel placed the individual's experience and behavior between the two-person level, for example the patient–physician interaction, and the nervous system (60).

Although the biopsychosocial model of medicine has in large part been practice-oriented, several interdisciplinary fields, such as psychoneuroendocrinology, psychoneuroimmunology, and, more recently, microbiota–gut–brain research, have been presented as research fields relevant to, expression of, or even as validation of the biopsychosocial model (61–65). However, the anthropologist Margot Lyon has pointed out that even though psychoneuroimmunology claims to embrace more than biology and puts forward the role of behavior in health and disease, “the problem of the representation of situatedness is the primary axis of tension in current research and writing in psychoneuroimmunology” ((66), p. 77). According to her, losing “the situatedness of understanding” may occur when representations in science are considered disconnected from context, and thus oppose the traditions of hermeneutics, emphasizing the importance of interpretation, and phenomenology ((66), p. 91). While admitting that efforts to think in terms of interactions between the immune, endocrine, and nervous system have the potential “to represent the organism simultaneously in its psychosocial and biological context,” Lyon also expressed the concern that, given the complexity of the psychosocial and biological as well as of the interactions between them, scientists are faced with “the problem of incalculable variables which cannot be experimentally eliminated” ((66), p. 91). According to her, the only ways to deal with these forms of complexities are either “through radical reduction, or through vast multidisciplinary studies which can bring many research strategies to bear on a single problem” ((66), p. 91).

### Cross-, Multi-, and Interdisciplinarity: Integration and Incompatibilities

In present-day academia, there are many calls and some incentives to engage in more multi- or interdisciplinary research and it is therefore worthwhile to get a better idea of what may be behind these terms. The social scientist Patricia Rosenfield in an article discussing the possibilities of “transdisciplinary research” to foster and further “the links between the health and social sciences” indicated that multidisciplinary research typically occurs when there is “a common problem or set of problems, [but] each discipline works independently and the results are usually brought together only at the end” ((67), p. 1351). In her perspective and in the case of interdisciplinary research, the “different disciplines use their techniques and skills [together] to address a common problem” ((67), p. 1351). Finally, according to Rosenfield, transdisciplinary research can lead to more complete understanding by encouraging scientists of several disciplines “to transcend their separate conceptual, theoretical, and methodological orientations in order to develop a shared approach to the research” and thus foster shared concepts ((67), p. 1351).

While the term multidisciplinary refers to less methodological and knowledge integration than that of interdisciplinary, it has been proposed that both can be grouped under the term cross-disciplinary ((68), p. 1938). The philosophers Michael O'Rourke and Stephen Crowly have made the case that philosophy can streamline the interaction of disciplines independently from the level of integration and have proposed a toolbox approach to do so (68). This approach is based on answers to two categories of questions, namely, “what we are like that we may know the world and what the world is like that we may know it” ((68), p. 1943). The confirmation section of the former category, for example, then invites scientists from different disciplines to indicate to what extent they agree or disagree with statements like “unreplicated results can be validated if confirmed by a combination of several different methods” ((68), p. 1952).

Interestingly, regarding health and disease, the philosopher and psychologist Derek Bolton together with the philosopher and neurosurgeon Grant Gillett has recently made a plea for more cross-disciplinarity arguing that “[t]here is simply too much going on for one disciplinary approach alone” ((69), pp. 100–101). According to these authors, part of the problem is that the disciplines that make up the biopsychosocial model slow down the ongoing “biopsychosocial/environmental transdisciplinary revamp across the life and human sciences” ((69), p. 101). One of the examples Bolton and Gillette provided to illustrate the need for such a transformation is relevant for sickness behavior. These authors indicated that reduced activity can be a consequence of illness or injury, which are experienced as pain and distress to make the point that “even these subjective experiences turn out to be thoroughly biopsychosocial” ((69), p. 117). Indeed, the pain field has long acknowledged that self-reports of pain also depend social factors and that pain-free injury can exist (70). Similarly, reduced activity during illness sickness or after injury also depends on psychological and sociological factors. Thus, it has been argued that understanding the functional limitations and reduction in the ability to engage in everyday activities of people with chronic osteoarthritis pain requires to take into account how people manage risks of falls and social isolation within their socio-environmental contexts (71). Most recently, income below the national US mean was found to be positively associated with sickness behavior in men as assessed with the Sickness questionnaire mentioned above (72). So, transcending disciplines may also require some deconstruction of disciplinary perspectives and approaches and getting acquainted with those of other disciplines.

However, transcending disciplinary traditions may not be that straightforward regarding illness/sickness behavior because, as pointed out by David Mechanic, “social scientists sought to depict the extraordinary variability of behavior, [whereas] physicians sought criteria to define ‘abnormal illness behavior”' ((18), p. 1208). Along these lines, it may also be telling that even in an attempt to unify the concept of illness behavior, Sirri and colleagues have proposed a framework that does not at all include the biological perspectives developed by Benjamin Hart and Robert Dantzer (27). This seems most of all to reflect mutual ignorance between disciplines employing seemingly related constructs. As indicated above, philosophy, medicine, sociology, psychology, and biology have all put forward ideas about health, disease, illness, and sickness in general and illness and sickness behavior in particular. However, this then begs the question of whether or not any attempt of integration should encompass all these ideas. In this context, it is important to keep in mind that (1) interdisciplinarity does not necessarily take place between high-level theories and can involve lower-level concepts or constructs, explanations, and methods (73) and (2) that different academic disciplines have different objectives. Concerning the latter point, it has been proposed that philosophers are typically interested in theories, whereas scientists care more about constructs and measures ((74), p. XXXI). Thus, philosophers study a theory's essential properties, for example by specifying necessary and sufficient conditions, while psychologists often use constructs that are thought to have observable and measurable expressions that can be assessed, for example by the use of questionnaires ((74), p. XXXI). Indeed for the philosopher Anna Alexandrova, constructs and measures should be closely related in the sense that “measures must reliably track constructs” ((74), p. XXXII).

These considerations seem to apply to health, disease, illness, and sickness behavior as well. On the one hand, philosophy has been highly active in trying to develop general theories of health and disease but has paid less attention to illness/sickness behaviors, not to mention on how to measure these. On the other hand, biology, psychology, and sociology have been more involved in trying to measure illness/sickness behavior without being that much concerned about a general theory. Thus, several constructs of illness and sickness behavior may have been put forward in sociology, psychology, and biology. Here it is important to keep in mind that constructs of the same name can be described differently depending on the domain. Indeed and as outlined above, illness behavior is described on the biomedical database PubMed as a “[c]oordinate set of non-specific behavioral responses to non-psychiatric illness” that “may include loss of appetite or libido, disinterest in activities of daily living or withdrawal from social interaction,” whereas on the APA database PsychInfo it refers to “behaviors, attitudes, and emotions exhibited by individuals during the course of a physical or mental illness.”

Furthermore, we have recently pointed out that under the same banner of sickness behavior, biology relates behavioral measures in animals while psychology mostly uses questionnaires addressing feelings in humans, even though behavioral observations are possible (75). Indeed, regarding the requirement that “measures must reliably track constructs” ((74), p. XXXII), several disciplines often follow different paths. However, beyond behavior as such, which can be observed both in animals and humans, there is a growing interest in the mental states that accompany sickness behaviors. However, biologists and veterinarians do not have a direct access to the mental states of animals, the way psychologists and physicians can rely on verbal report of mental states in humans. Nevertheless, concluding as to the mental state of hunger or appetite when animals ingest more food than after a period of restricted access or to those of pain or nausea based on their facial expressions (76–78) seems rather straightforward. The critical point here is the interpretation of the behavioral observations in the process of making inferences about the mental states of animals. For example, rodent tests based on the time spent in open well-lit spaces or immobile in inescapable situations to assess anxiety or depression constructs have been criticized (79–82). These examples suggest that different research traditions can lead to diverse constructs even when the latter go by under similar names.

### Stress Test for Interdisciplinarity

A historic example of diverse constructs used in different research traditions that have the same name is stress. The Hongro-Canadian physician Hans Selye is often credited for having given contents to the term stress in the life sciences. Indeed, he has proposed to coin his general adaptation syndrome to different adverse situations, stress (83), and to include infections under “systemic stress” ((84), p. 190). However, Selye has also attempted to progressively provide terminological clarity and to reserve the term stress to biological responses and that of stressors to the diverse stimuli and to distinguish between eustress and distress (85). Interestingly, early on George Engel made it clear that chemical, physiological, psychological, and social means can be mobilized to cope with stress(ors) and illustrated this with bacterial infection of a human being as an example (4). In this case, both a local inflammatory reaction and an overall modification of physiology can be observed as part of the host defense ((4), p. 55). However, Engel emphasized that “we also see psychologic defenses,” such as “increased dependence [and] withdrawal of interest in the outer world” ((4), p. 55). Finally, he pointed out that, in addition, social and institutional resources can be mobilized (4). Based on this example, Engel seemed to consider biological, psychological, and sociological responses as different ways to deal with the stress(or) of an infection. In his later work, however, he also stated that “the need of the patient is to be relieved of ‘distress' rightly or wrongly attributed to ‘illness”' ((86), p. 102), suggesting that distress corresponds to the psychological state of an ill individual. Within the context of sociology, several scholars have studied “families under stress” after the Second World War (87, 88). While the sociologist David Mechanic judged that “The concept of stress has not been adequately or precisely defined in the behavioral sciences,” he proposed that is a state related to “anxiety, discomfort, emotional tension, and difficulty in adjustment” ((89), p. 51). He mostly referred to “perceived stress” and saw stress as a psychological state that can modulate illness behavior. Indeed, Mechanic found that individuals with reported “high stress,” as measured by frequency of loneliness and nervousness, were significantly more likely to use medical facilities than persons with lesser “stress” ((17), pp. 191–192).

The philosopher Wim van der Steen considered in 1993 that in the beginning the investigation of stress took place in separate disciplines without much interaction (90). He credited Selye's physiological research for having “discovered that many adverse conditions produced the same kind of physiological responses in organisms,” which was then “called the stress response” ((90), p. 263). However, Van der Steen also remarked that around the same time psychologists studying stress “were primarily interested in stimuli and in internal states of organisms, psychologically characterized” ((90), p. 263). Thus, physiologists have used the term stress for response whereas psychologists have employed the same to refer to stimuli and internal states. For Van der Steen, this is confusing and counterproductive given that independent descriptions of stimuli and responses are required for sound empirical research to take place (90).

If interdisciplinary research fields such as psychoneuroendocrinology and psychoneuroimmunology have over the past 25 years widely employed the terms stressor and stress response and have often distinguished physiological from psychological stress, the danger of confusing stimuli, internal states, and responses may be present for more recent interdisciplinary domains less aware of this historic debate. With respect to illness/sickness, one could specify that bacterial LPS fragments constitute a physiological stressor for animals and illness/sickness behavior is part of the stress response. To what the (di)stressed state of an infected organism (or experimental model thereof) corresponds is still a matter of active research, but anxiety may be a good candidate (49). However, although the very name of illness/sickness behavior seems to indicate a response of the organism, the risk is still present that various disciplines consider this differently. For example, the sickness behavior questionnaire mentioned above contains mainly questions about how the subject feels and seems to address less how the subject engages or not in daily activities (50).

### Motivation as a Bridge Between Disciplines?

Interestingly, the notion of motivation has been mentioned in the context of illness/sickness behavior in sociology, biology, and psychology. Thus, the sociologist Talcott Parsons remarked that illness was motivated, but considered it a kind of “deviant behavior” ((10), p. 285). This latter qualification should probably be seen in the light of Parsons' hypothesis that the advantages and relief of responsibilities for the sick could constitute a “secondary gain” that can motivate an individual to become or remain sick ((10), p. 437). Several decades later, David Mechanic seemed to put this in a broader and different perspective by indicating that clinical practice learns that there are also many individuals, who, even when afflicted with disease, manage to work or have other activities precisely because they are motivated (18). Interestingly, neither the illness behavior questionnaire nor a recent article proposing to unify illness behavior mentioned motivation (23, 27). The notion of motivation can, however, be encountered regarding health-related choices, for example when it comes to those of former smokers. In this context, maintenance of non-smoking has been proposed to involve “motivational … mechanisms of self-change” ((91), p. 943). So despite the fact that motivation was an important topic to position the sick role and illness behavior concepts, it seems to be considered in different contexts in present-day medical sociology, even though its study in relation to medically unexplained chronic symptoms, such as pain and fatigue, will likely continue to prove to be relevant, for example in post-Covid-19 infection.

Benjamin Hart clearly worked from an evolutionary background for sickness behavior when he proposed that an infected animal that is sleepy or inactive “is less motivated to move about using energy that could fuel metabolic increases associated with fever” ((16), p. 129). Although a veterinarian by training, Robert Dantzer referred to psychology to define motivation “as a central state that reorganizes perception and action” using fear as an illustration and to emphasize that states of motivation allow to dissociate perception and action depending on the circumstances and do therefore not given rise to a fixed behavioral response ((92), p. 14). This specification served as rationale for the work of Aubert cited above showing that injection of a dose of LPS injection to lactating mice did not result in nest building when pups were placed throughout the cage at 20°C, but did induce nest building after pups were dispersed at 4°C (92). Thus, Dantzer concluded that “sickness behavior appears to be the expression of a central motivational state” ((92), p. 7, p. 20). It remained to be seen for him, however, if the sickness motivational system can account for all of the responses of an organism after activation of the innate immune system or if it “must be included in another more basic motivational system, such as the pain defense system” ((93), p. 155). One aspect of animal sickness behavior that can be considered from a motivational standpoint is behavioral expression in a social context. Interestingly, reduced agonistic behaviors have been reported in dominant mice, but not in submissive animals, after LPS administration, a finding that can be interpreted to indicate “that the expression of sickness-associated behaviors relies on a motivational reorganization and change in priorities that should differ according to social rank” ((94), pp. 114–115).

A classic theme in health psychology is that of coping with chronic disease. In this context, it is considered that social support can promote the adoption of adequate coping strategies by improving understanding and increasing motivation (95). It is important to emphasize here that coping can involve “approaching or avoiding the demands of chronic disease” and that the positions adopted by individual on this continuum involve motivations (95). More recently and regarding the acute disease model of LPS administration to young healthy volunteers, Lasselin and colleagues have reported an increased motivation to opt for the “high-effort/high-reward mode of response, but only when the probability to win [a monetary reward] was the highest” ((49), p. 801). Others have promoted the idea of a so-called behavioral immune system that represents “a unique motivational system” and is closely associated with disgust ((96), p. 251). Thus, infections result in the emotion of disgust, which, in turn, leads to motivated “behavioral avoidance” ((97), p. 6). Interestingly, these considerations have been put forward within the context of “an evolutionary approach to socio-ecological psychology” ((97), p. 6). As indicated above, some psychologists have recently shown how gait and facial cues allow subjects to recognize a sick person (44, 51). It would thus be interesting to study if and how these social cues affect motivation.

## Conclusion

On the one hand, many of the interdisciplinary initiatives involving sociology and medicine, like social medicine, the sociology of health and disease, and the biopsychosocial model of medicine, have emphasized the importance of a holistic approach and of considering multiple factors (6, 98). On the other hand, numerous interdisciplinary research efforts mobilizing psychology and biology have been done within the framework of evolution. This then allows, following Ernst Mayr, to distinguish how and why questions about, proximate causation relative to causal mechanisms operating within the life of the organism and ultimate causation regarding adaptation or drift over evolutionary time (99). When it comes to the possibility of an interdisciplinary approach of illness/sickness behavior, this means that multiple causes would need to be considered from the perspective of the organism. It seems that the notions of stressor, between infection as a physiological stressor and dominance as a psychosocial stressors, and of motivation as a state offer opportunities for biology, medicine, psychology, and sociology to communicate and collaborate to further our understanding of illness/sickness behaviors in particular those accompanying chronic conditions. So it seems that interdisciplinary research on illness/sickness behavior between biology, psychology, and sociology with the aim of “integration of the constructs, data and explanations” is possible ((100), p. 129). However, this will require “coordinated pluralism” (100) with regard to constructs, methods, and findings and awareness of differences in perspectives, for example between a focus on acute and chronic sickness behavior.

## Author Contributions

JPK conceived of, wrote, and edited this work.

## Conflict of Interest

The author declares that the research was conducted in the absence of any commercial or financial relationships that could be construed as a potential conflict of interest.
